# Recruiting Resident Participants from Nursing Homes During the COVID Pandemic: Challenges and Solutions

**DOI:** 10.1093/geroni/igab046.3526

**Published:** 2021-12-17

**Authors:** Leena Almasri, Barbara Carlson, Julie Myers, Rebecca Koszalinski, Melissa Franklin, Alysa Kelsh, Diana Sturdevant

**Affiliations:** 1 University of Oklahoma Health Sciences Center, Oklahoma City, Oklahoma, United States; 2 University of Oklahoma HSC, Oklahoma City, Oklahoma, United States

## Abstract

Recruiting nursing home residents as participants in research is challenging. In early 2021, Covid-19 cases rose rapidly in nursing homes, prompting the rapid deployment of infectious disease protocols and ultimately, facility lockdowns to control the spread of the virus. By September, 2020, many research projects were delayed or cancelled, and future research was jeopardized. During this period, as well as prior to and after the administration of the COVID vaccine, we enrolled residents in a complex protocol involving administration of two Shingles vaccines (0- and 90 days) and three separate blood samples. Here, we present the strategies we used to recruit 216 residents, from 23 homes, over a 9-month period. We faced many challenges. Our research staff faced weekly COVID-19 antigen tests prior to entering the facility, adhering to strict protocols on travel, as well as packaging of materials that entered and left the facility. N95 masks and face shields further made it difficult to communicate with residents. For homes, COVID protocols required residents to be transported to specified areas to meet with research staff. Daily monitoring of COVID and Shingrix vaccine symptoms became part of daily care. To minimize resident harm and interruption of workflow in nursing homes, we utilized principles of stakeholder engagement, healthcare leadership, infectious disease/immunology, and staff (research and nursing homes) empowerment. In the face of crisis, like the COVID-19 pandemic, we have gained the trust and commitment of these facilities; thus, establishing a sustainable partnership that is prepared for what comes next.

